# Why ethnography matters in global health: The case of the traditional birth attendant

**DOI:** 10.7189/jogh.07.020302

**Published:** 2017-12

**Authors:** Margaret MacDonald

**Affiliations:** Department of Anthropology, York University, Toronto, Canada

The representative from the foreign aid agency, a woman with whom I had crossed paths several times in the small town of Mangochi in southern Malawi, banged her fist on the table. “How can we get them to distribute condoms? They should be distributing condoms!” She was referring to the traditional birth attendants (TBAs) in the Monkey Bay Safe Motherhood project that we were there in the meeting to discuss. She envisioned them as ideal community actors in the HIV prevention project she worked for. I had been working in Monkey Bay for the past three months evaluating a Safe Motherhood project. I was also a graduate student in anthropology at the time and therefore inclined to take an ethnographic approach to my task. I knew the TBAs wouldn’t distribute condoms. I had been to their homes to talk to them about their work and had asked them as politely as it is possible to do so how they felt about distributing condoms. “Oh no,” they invariably told me. “We don’t do that. It’s not our place to talk to men about that.” I had also watched as they slid the ‘Safe Birth Kits’ they had received as part of their training out from under beds or curtained closets, opened the lids to reveal lengths of condoms still in their packages, closed the kits again and slid them carefully back into place. Imagined by a foreign aid agency to be working in the realm of ‘reproductive and sexual health’, these TBAs refused. Their work was attending births. They did not fail to fit the bill, they refused it.

Many who work as practitioners in global health could recount similar stories. They might include them in project reports to add texture or to illustrate a point, but anthropologists consider such stories ethnographic data that, with proper context and analysis, constitute reliable and robust knowledge. In this article I will argue that ethnography matters in global health as an essential component of interdisciplinary efforts to understand and address complex global health problems.

But first let me continue the ethnographic story. The information I shared in the meeting room in Mangochi – that TBAs were highly unlikely to ever distribute condoms within their communities – may have gone one of three ways. It may have been ignored and the HIV prevention project may have continued to press for community based condom distribution by TBAs. It may have been noted as a ‘cultural barrier’ to achieving the goals of the project and become the object of a new set of strategies – adding a component on the importance of condom distribution in the next round of TBA training sessions and refresher courses, for example. Or the information I shared may have been taken as new ‘evidence’ that provoked a rethinking of the means by which to achieve project goals. I will never know. I wrote up my report on the Safe Motherhood project which included the recommendation that TBAs should not be pressed to distribute condoms; it was not a cultural barrier to be overcome by education or training but a faulty assumption about the scope of practice of these specific TBAs in this particular setting. I would venture to say that trying to get TBAs in rural Malawi to distribute condoms did not work for ‘cultural reasons’ – but only on the understanding that these ‘cultural reasons’ lay with the foreign aid agency that suggested it as a strategy in the first place. The idea that cultural barriers to change must be combatted for interventions to succeed is a staple of global development thinking. But global health communities have cultures too: sets of assumptions, taxonomies, and rationales that just as surely contribute to the outcome of local health projects.

## WHY ETHNOGRAPHY MATTERS

*Ethnography matters for contemporary societies…This claim derives from the very activity of the ethnographer – a presence both involved and detached, inscribed in the instant and over time, allowing precise descriptions and multiple perspectives, thus providing a distinctive understanding of the world that deserves to be shared* [1].

Ethnography is the hallmark of the discipline of anthropology. It involves following the everyday lives of people over long periods of time. Anthropologists immerse themselves for months and years in the communities they seek to understand, learning local languages and living to a great extent as local people do. The kinds of communities that anthropologists study are broader now than in the past. A rural village, a busy urban health clinic, or a group of foreign aid workers in their field offices: these are all ethnographic sites for anthropologists. Though lives may be framed by big events and issues, it is the quotidian that is the essence of ethnography. Even life in exceptional times and situations – conflict zones, prisons, refugee camps, epidemics – develops patterns and ways of being. At the same time, ethnographers approach communities and cultures as dynamic entities with the capacity to change – whether the change is initiated from within or without.

The point I want to stress here is that ethnographers seeks to make visible the practical and moral worlds and actions of individuals and cultural groups, but not in isolation of history and politics. Indeed, critical ethnographers excel at illuminating the connections between the micro and macro levels. Ultimately, the goal of ethnography is to offer better accounts of social phenomena than one can from a distance, from secondary accounts, or from rapid appraisals. By this method, ethnographers produce knowledge that is robust and reliable, if not reproducible.

## THE CASE OF TRADITIONAL BIRTH ATTENDANTS IN GLOBAL MATERNAL HEALTH

To illustrate how ethnography matters in global health, I will return to the case of the Traditional Birth Attendant deployed in maternal health interventions. This requires a bit of history. The first global initiative to address the problem of maternal mortality was the Safe Motherhood Initiative (SMI), launched in 1987 by the World Health Organisation (WHO), the World Bank, and the United Nations Population Fund (UNFPA). At that time the global maternal mortality rate was estimated at 500 000 a year – vast majority of deaths occurring in the global south. The stated goal of the initiative was to reduce this number by half by the year 2000, through a package of upgrades to health systems, personnel, and family planning activities. One of the key interventions was the training of birth attendants to better cope with births at the community level and to identify and refer women at risk to health facilities. UNICEF and other organisations had been training TBAs since the 1970s and it appeared a progressive move to acknowledge traditional medical cultures and incorporate local practitioners in a more systematic way.

But a little more than a decade after its launch, the TBA component of the SMI was reviewed, deemed a failure, and side–lined in favour of increasing the number of ‘skilled birth attendants’ globally [2]. The decision caused controversy at the time, with many practitioners and researchers arguing that the work of TBAs had not been properly understood or evaluated [3]. What if ethnographic data on birth attendance had mattered in this effort from the beginning? Would we have better understood what roles TBAs were already playing and what roles they could be expected to play? On the basis of such knowledge of local logic and practice concerning pregnancy and birth, could interventions have been designed and launched to appropriately train and support TBAs to make a difference?

As early as 1978 anthropologist Brigitte Jordan had described ineffective and inappropriate methods used in the training of Maya midwives in the Yucatan: didactic rather hands–on learning, lack of cultural sensitivity by trainers towards *parteras,* and no follow–up [4]. In addition to documenting pedagogical problems with training and supervision, some anthropologists began to call into question the very notion of a TBA. In Nepal, for example, there was no local equivalent of the TBA. The women who came forward to receive training had no special experience or expertise with childbirth; TBAs had to be “invented” to fit into SMI activities [5]. Similarly, in Tanzania among Sukuma communities, there was no distinct tradition of midwifery and many women gave birth with female relatives or alone. Some 30% of women identified and trained as TBAs by a local SMI project had never attended a birth before [6].

**Figure:**
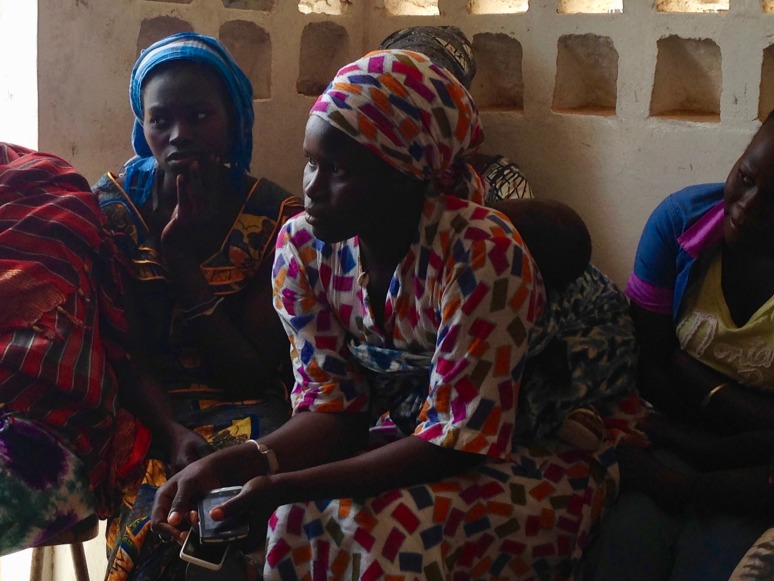
Photo: from author’s own collection (used with permission).

Anthropologists identified other problems as well. Guidelines for identifying women to be trained as TBAs did not consider the reality that factors such as ethnicity, language, religion and kinship can be more important to women and their families than training in the choice of a birth attendant [7]. In Malawi I observed that many nurse–midwives resented being drafted into the role of TBA liaison by SMI policy. Yet in some places in some ways TBAs were working effectively – able to identify women with complications and refer them to health facilities, for example. Ethnographic data could have provided insight into why.

Not only were TBAs deemed failures in the reduction of maternal deaths, they also came to be seen as obstacles to development for their failure to take on these new roles designated for them by SMI policy. Anthropologist Denise Roth Allen sums it up well:

*When women who have had no experience delivering babies are somehow turned into traditional birth attendants in the span of a ten–day training course, it is hardly surprising that TBA training programs have not produced the results policy makers and program planners originally intended; nor is it surprising that pregnant women residing in rural areas perceive some of these TBAs as risks rather than as sources of labor support when birth is imminent *[6], p. 115.

The irony is that the SMI had tried to respect and incorporate local tradition rather than steamroll it. But the local imagined by the SMI was paradoxically too general; the TBA was imagined as a universal type. Significantly, she was also imagined as being able to improve maternal health without the aid of a functioning health care system in many places and in the midst of an HIV epidemic in others. Some critics charged that TBAs had been scapegoated.

A recent article in this journal has called for the ‘return of the traditional birth attendant’ arguing that it makes pragmatic sense given that so many women in low resource settings still do not have access to adequate health services and that “for many women for a range of reasons TBAs are preferable to hospital care” [8]. As maternal mortality remains high on the global health agenda and the search for innovation in research and interventions continues to scale up, ethnography matters more than ever. It adds context and history to the understanding of present day health challenges for people and populations around the world rather than seeing them as locally made and isolated problems of underdevelopment or culture. It can illuminate the logic and rationale of people and communities at the local level and see them in their particularity, not in their global otherness. It can help explain why some interventions fail and others succeed. It can help identify modes of change that make sense not universally but in a given context.

In sum, ethnography matters in global health because it produces distinctive, locally grounded knowledge that can contribute to the multi–disciplinary and inter–sectoral effort to create effective solutions to pressing and persistent global health problems. Why global health culture has not embraced ethnography is a question ripe for discussion. Certainly, the dominance of quantitative research and the trend towards accountability metrics in global health is a factor, even though qualitative methodologies are better able to capture the social relations of health care so fundamental to the changes in knowledge and behaviour most global health interventions seek [9,10]. There are several directions we might take to change the marginal status of ethnographic knowledge in global health. Anthropologists can continue to conduct critical ethnographic studies of global health policy–making circles to offer insight into these ‘epistemic communities’ in terms of the knowledge and values they share and the fiscal, ideological and political pressures they must abide. Collaborative research is another way forward, taking on the challenges of cross–disciplinary communication in both the conduct of research and writing [11]. We can also disseminate the results of our research to new audiences. However we move forward, making the case for ethnography is an important part of the work of anthropologists engaged in global health research and practice today.
